# Standard Percutaneous Transluminal Angioplasty Versus Intravascular Lithotripsy to Facilitate Trans-Femoral Transcatheter Aortic Valve Implantation in Patients with Aortic Stenosis and Severe Peripheral Arterial Disease

**DOI:** 10.3390/jcm14176335

**Published:** 2025-09-08

**Authors:** David Belkin, Tamir Bental, Tullio Palmerini, Ran Kornowski, Pablo Codner

**Affiliations:** 1 The Faculty of Medicine, Tel Aviv University, Tel Aviv 6997801, Israel; belkin@mail.tau.ac.il (D.B.); tamirb@clalit.org.il (T.B.); ran.kornowski@gmail.com (R.K.); 2 Rabin Medical Center, Petah Tikva 49100, Israel; 3 Cardiology Unit, Cardiac Thoracic and Vascular Department, IRCCS Azienda Ospedaliero-Universitaria di Bologna, 40138 Bologna, Italy; tulliopalmerini@hotmail.com

**Keywords:** TAVI, aortic stenosis, peripheral arterial disease, intravascular lithotripsy, percutaneous transluminal angioplasty

## Abstract

**Background/Objectives**: The optimal method to facilitate femoral access in patients with aortic stenosis and severe peripheral arterial disease (PAD) undergoing transcatheter aortic valve implantation (TAVI) remains unclear. This study compared the safety and efficacy of percutaneous transluminal angioplasty (PTA) versus Shockwave^®^ intravascular lithotripsy (IVL) in patients with severe PAD undergoing TAVI via the trans-femoral route enrolled in the Hostile TAVI registry trial. **Methods**: Patients with severe PAD from 28 international centers were enrolled in the registry. This sub-study analyzed patients who underwent transfemoral TAVI facilitated by PTA (*n* = 352) or IVL (*n* = 166). Primary endpoints included rates of survival, major vascular complications, and major bleeding. Outcomes were also stratified according to the severity of PAD using the novel Hostile risk score. **Results**: Patients in the PTA group were older and had lower rates of prior stroke/TIA. All-cause mortality at 3 years was similar between PTA and IVL groups (34.9% vs. 38.6%; *p* = 0.27, respectively). However, IVL was associated with fewer major vascular complications (21.7% vs. 13.3%; *p* = 0.033, respectively), less major bleeding (14.0% vs. 7.0%; *p* = 0.024, respectively), and shorter hospital stays (7.06 ± 6.69 vs. 4.29 ± 4.78 days; *p* < 0.001; 95% CI: 1.63–3.91, respectively). Cox regression analysis showed that at low (≤8.5) Hostile Scores, PTA was associated with higher rates of major vascular complications and major bleeding than IVL. **Conclusions**: In patients with aortic stenosis and severe PAD undergoing TAVI via the transfemoral route, IVL is safer than PTA, with fewer vascular and bleeding complications but similar intermediate-term survival.

## 1. Introduction

Transcatheter aortic valve implantation (TAVI) is a well-established treatment for patients with severe symptomatic aortic stenosis (AS) [1,2]. Severe aortic stenosis typically presents with progressive symptoms including dyspnea on exertion, angina, and syncope, reflecting the hemodynamic burden imposed by left ventricular outflow obstruction. In some cases, patients may present acutely with heart failure or cardiogenic shock, underscoring the urgency of prompt diagnosis and intervention. Timely treatment is essential, as untreated symptomatic aortic stenosis is associated with a markedly increased risk of mortality [3]. Most patients (90–95%) are treated via transfemoral access (TFA) [4]. The remaining minority typically have severe peripheral arterial disease (PAD) that precludes TFA and are instead treated via alternative (non-femoral) approaches, including transthoracic (TTA) and transaxillary (TAA) [5]. Percutaneous transluminal angioplasty (PTA) and intravascular lithotripsy (IVL) are techniques that allow for TFA approach in patients with severe PAD receiving TAVI [6,7]. However, the relative safety and efficacy of PTA versus IVL in this group of patients has never been investigated.

This sub-study utilized the Hostile registry, a multicenter, international study of patients with PAD undergoing TAVI, to perform a comparative analysis of those treated with either PTA or IVL [8]. As part of this registry, the Hostile Score was developed as a novel anatomical risk stratification tool to predict procedural complexity and vascular complications in this patient population. It incorporates key anatomical features of the iliofemoral vasculature, including vessel calcification, minimal lumen diameter, eccentricity, and tortuosity, all derived from preprocedural CT imaging. Each component contributes to an overall score that reflects the severity of femoral access difficulty. In the original Hostile registry study, higher scores were associated with significantly increased rates of vascular complications, procedural failure, and adverse clinical outcomes, thus offering a valuable tool for access route planning and risk prediction in this challenging population.

## 2. Methods

The Hostile registry included 1707 patients with PAD and ‘hostile’ femoral access undergoing TAVI in 28 international medical centers [8]. Patients were prospectively recruited between June 2021 and February 2022 after local ethics committee approval and written informed consent. Additionally, data from consecutive patients at participating centers were retrospectively entered into the registry under the governance of local ethics committees for use of de-identified data.

These patients were divided into subgroups based on the access approach (TFA, *n* = 518; TTA, *n* = 642; and TAA, *n* = 547). In the present study, we considered TFA-treated patients only, who were further stratified according to type of peripheral vascular intervention: PTA versus IVL.

Primary outcomes were: (1) major bleeding, (2) major vascular complications, and (3) all-cause mortality. Secondary outcomes included: (1) stroke/TIA, (2) a composite of all-cause mortality, stroke/TIA, and major vascular complications, and (3) length of hospital stay. Outcomes were stratified according to the severity of PAD using the novel Hostile risk score [8] and adjusted for age and prior stroke/TIA. Endpoints were adjudicated according to Valve Academic Research Consortium (VARC)-3 criteria [9]. Composite endpoints were decomposed into their individual components: all-cause mortality, 30-day major vascular complications, and stroke/TIA; each was analyzed separately with event rates and 95% confidence intervals.

Data are presented as mean ± standard deviation for continuous variables. Continuous variables were compared using the Student’s *t*-test, and categorical variables were compared using the chi-square test or Fisher’s exact test, as appropriate. All *p*-values were adjusted for age and prior stroke/TIA using Cox regression analysis. All tests were 2-tailed, and a *p*-value < 0.05 was considered significant. Event-free survival of PTA versus IVL access method was analyzed using Kaplan–Meier survival curves with a log-rank test. For analysis of the Hostile Score [8], patients were grouped using binning by Hostile Score and a multivariate Cox regression analysis was used to define the significance of the score. We also performed additional regression models using the Hostile Score as a continuous variable. Multivariable logistic regression models were expanded to include procedural access characteristics such as access type (surgical vs. percutaneous) and closure device. Furthermore, a gender-stratified exploratory analysis of 30-day vascular complications, stroke/TIA, and all-cause mortality in both treatment arms (PTA and IVL) was performed.

## 3. Results

Among the 518 TFA-treated patients included in the Hostile registry, 352 (67.9%) were treated with PTA and 166 (32.1%) were treated with IVL. Baseline clinical characteristics of patients treated with PTA versus IVL are shown in Table 1.

Patients in the PTA group were more commonly treated with surgical cutdown in comparison to the IVL group (13.6% vs. 5.4%, *p* = 0.003, respectively) (Table 2).

The self-expanding Medtronic Evolut/CoreValve and the balloon-expandable Sapien transcatheter heart valves (THVs) were the most used THVs in both groups (82.7% vs. 77.2% in the PTA vs. the IVL group, respectively) (Table 2).

**Figure 1:**
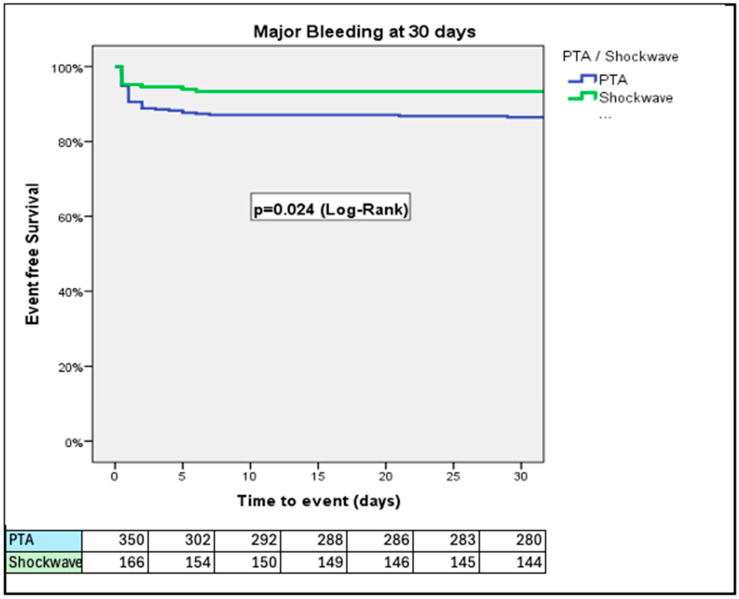
Rates of VARC-3 defined major bleeding, adjusted for age and prior stroke/TIA. PTA patients had a higher rate of major bleeding in comparison to the IVL group.

**Figure 2:**
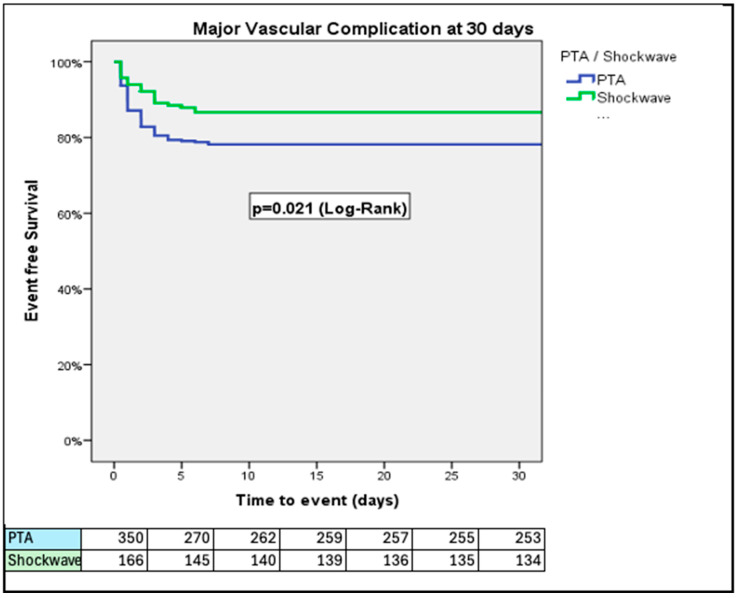
Rates of VARC-3 defined major vascular complications, adjusted for age and prior stroke/TIA. PTA patients had a higher rate of major vascular complications in comparison to the IVL group.

**Figure 3:**
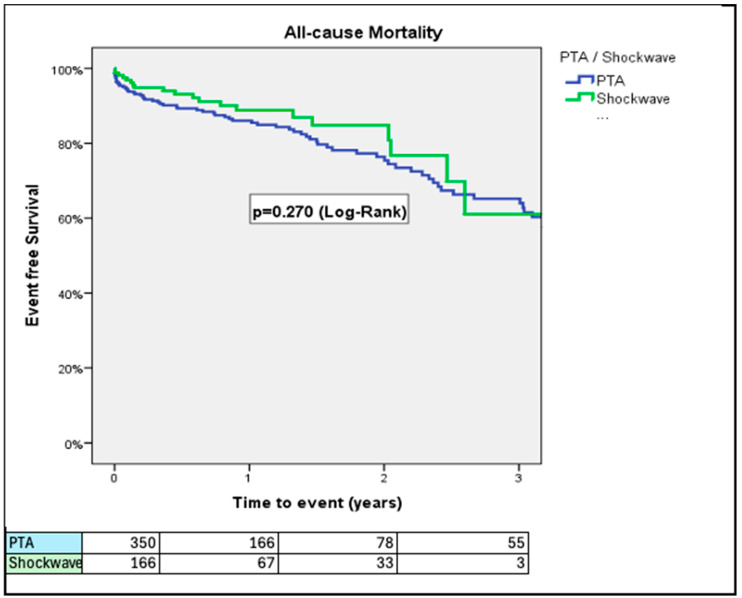
Rates of all-cause mortality, adjusted for age and prior stroke/TIA. Rates of all-cause mortality were similar over a 3-year period.

At 30 days, PTA patients had higher rates of VARC-3 defined major bleeding (14.0% vs. 7.0%; *p* = 0.024) and major vascular complications (21.7% vs. 13.3%; *p* = 0.033) than IVL patients (Figure 1 and Figure 2). The rates of all-cause mortality were similar over a 3-year period (34.9% vs. 38.6%; *p* = 0.27, for PTA vs. IVL, respectively) (Figure 3).

**Figure 4:**
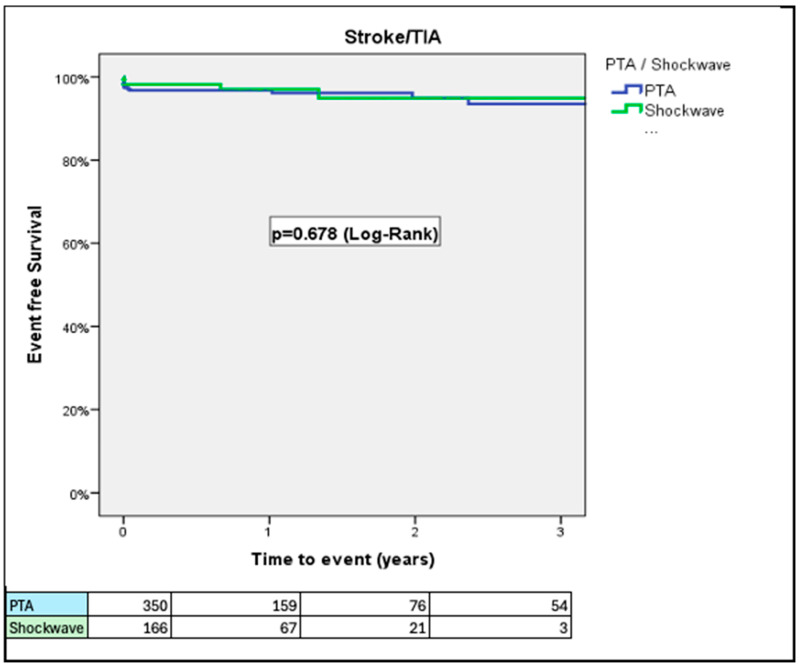
Rates of stroke/TIA, adjusted for age and prior stroke/TIA. Rates of stroke/TIA were similar over a 3-year period.

**Figure 5:**
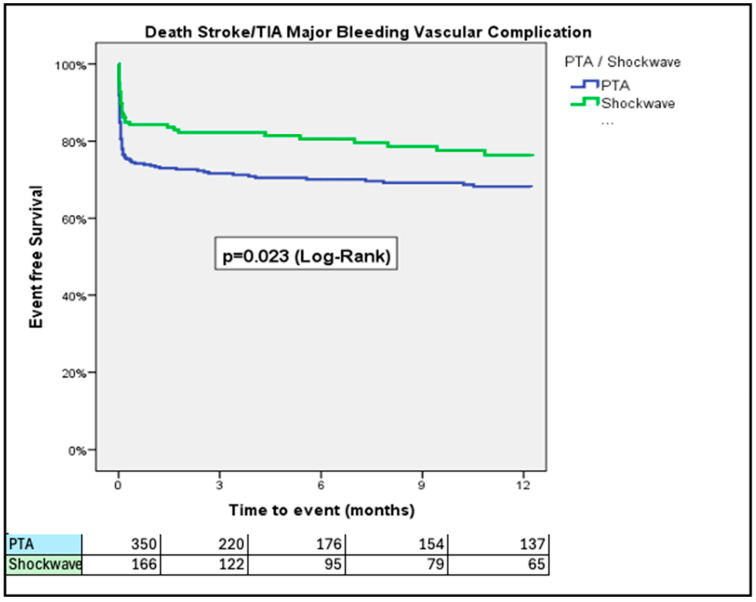
Rates of the composite of all-cause mortality, stroke/TIA, and major vascular complications, adjusted for age and prior stroke/TIA. PTA patients had a higher rate of the composite of all-cause mortality, stroke/TIA, and major vascular complications in comparison to the IVL group.

**Figure A1:**
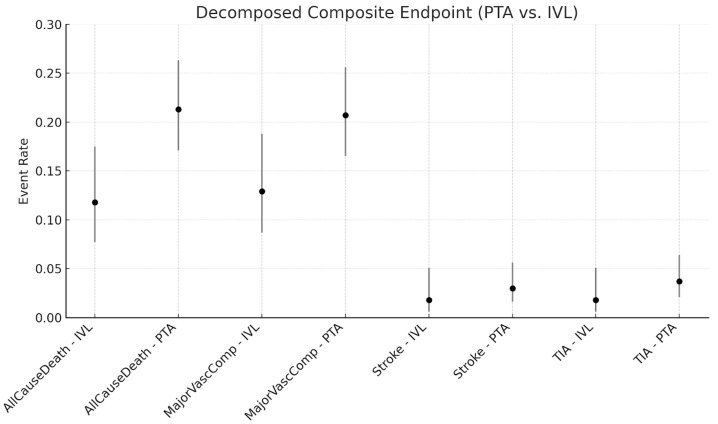
Event rates with 95% confidence intervals for individual components of the composite endpoint by treatment group (PTA vs. IVL).

Rates of stroke/TIA did not differ between the two groups (Figure 4). The rate of the composite of all-cause mortality, stroke/TIA, and major vascular complications was significantly higher in patients treated with PTA in comparison to patients treated with IVL at 1-year follow up (32.1% vs. 23.9%, *p* = 0.041, respectively) (Figure 5). This difference was driven by higher rates of major vascular complications in the PTA group. Length of hospital stay after the index procedure was longer in the PTA versus the IVL group (7.06 ± 6.69 vs. 4.29 ± 4.78 days; *p* < 0.001; 95% CI: 1.63–3.91, respectively). IVL was associated with lower event rates across all components of the composite endpoint, including all-cause mortality (11.8% vs. 21.3%), major vascular complications (12.9% vs. 20.7%), and stroke/TIA (1.8% vs. 3.5%) compared to PTA (Figure A1).

**Figure 6:**
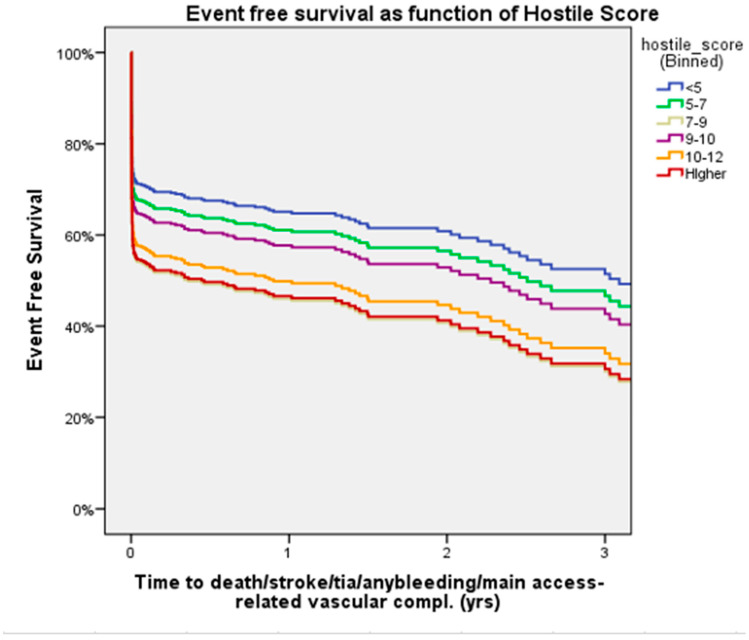
Event-free (all-cause mortality, major bleeding, major vascular complications, or stroke/TIA) survival as a function of the Hostile Score, adjusted for age and prior stroke/TIA. A higher Hostile Score correlated with a greater risk of all-cause mortality, major bleeding, major vascular complications, and stroke/TIA, irrespective of PTA or IVL.

Cox regression analysis showed that the Hostile Score is an independent predictor of all-cause mortality, major bleeding, major vascular complications, and stroke/TIA, irrespective of treatment with PTA or IVL (Figure 6). Neither access type nor closure device was significantly associated with major vascular complications; treatment with IVL remained associated with a lower, though borderline non-significant, risk of complications (Table A1).

**Figure 7:**
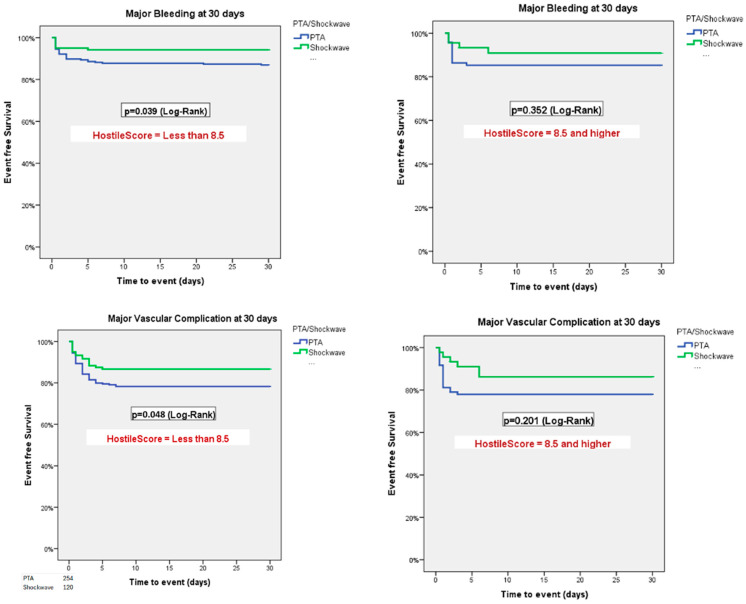
Rates of VARC-3 defined major bleeding and major vascular complications at 30 days stratified by Hostile Score ≤ 8.5 versus > 8.5, adjusted for age and prior stroke/TIA. In patients with a Hostile Score ≤ 8.5, PTA was associated with higher rates of VARC-3 major vascular complications and bleeding. No differences were observed between PTA and IVL in those with a Hostile Score > 8.5.

**Figure A2:**
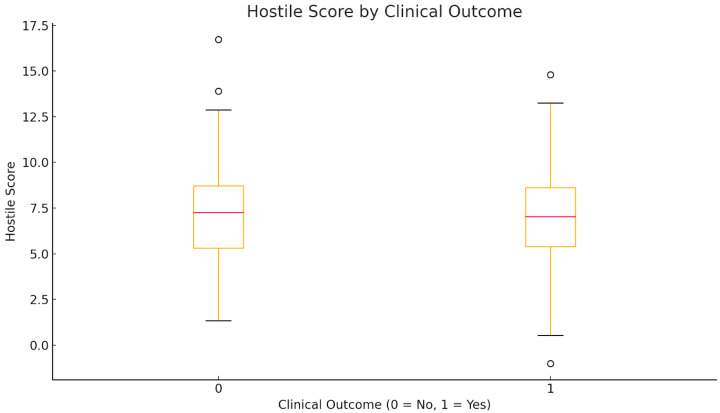
Hostile Score distribution by clinical outcome (0 = No event, 1 = Event).

Patients were stratified according to Hostile Score (≤8.5 vs. >8.5). In patients with a Hostile Score ≤ 8.5, PTA was associated with higher rates of VARC-3 major bleeding (*p* = 0.039) and major vascular complications (*p* = 0.048). No differences were observed between PTA and IVL in those with a Hostile Score > 8.5 (Figure 7). Additional regression models using the Hostile Score as a continuous variable found no significant association with 30-day vascular complications or mortality (Figure A2).

**Figure A3:**
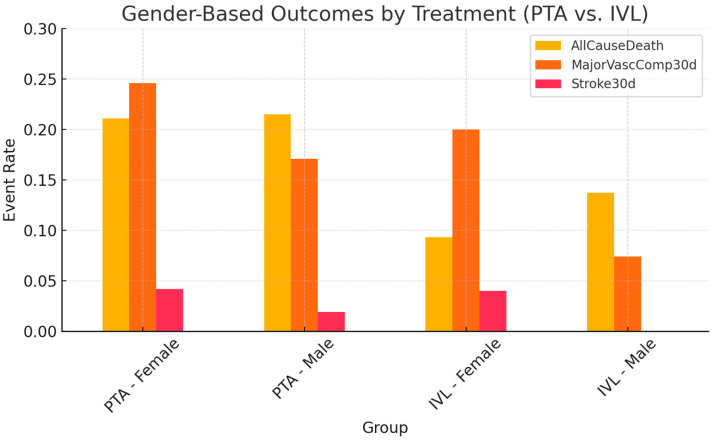
Gender-stratified event rates for all-cause mortality, major vascular complications, and stroke at 30 days by treatment type (PTA vs. IVL).

Women treated with IVL had the lowest event rates across all outcomes, while men treated with PTA had the highest; this included a major vascular complication rate of 7.4% in IVL-treated women versus 24.6% in PTA-treated men (Figure A3).

## 4. Discussion

In this sub-study of the Hostile registry, we investigated outcomes in patients with severe PAD undergoing TAVI via transfemoral access treated with either PTA or IVL. Our main findings were as follows: (1) At 30 days, PTA patients had higher rates of major bleeding and major vascular complications than IVL patients; (2) at 3 years, rates of all-cause mortality were similar between PTA and IVL patients; (3) at 3 years, rates of stroke/TIA were similar between PTA and IVL patients; (4) at 1 year, rates of the composite of all-cause mortality, stroke/TIA, and major vascular complications were higher in patients treated with PTA than those treated with IVL; (5) length of hospital stay after the index procedure was longer in the PTA than in the IVL group; (6) the Hostile Score is an independent predictor of all-cause mortality, major bleeding, major vascular complications, and stroke/TIA, irrespective of treatment with PTA or IVL; and (7) at a low (≤8.5) Hostile Score, patients treated with PTA had a higher risk of major bleeding and major vascular complications than their counterparts treated with IVL.

When TAVI was first introduced, major vascular complications were frequent, even in patients without PAD [10]. The PARTNER trial reported major vascular complications in 15.3% of patients [11], and a meta-analysis of 3519 patients treated from 2010 to 2017 reported a major vascular complication rate of 11.9% [12]. However, in recent years, with the advancement and implementation of ultrasound-guided access, the systematic use of CT angiography for access planning, improvements in delivery systems, and an overall increase in operator experience, rates of vascular complications amongst non-PAD patients undergoing TAVI have dropped dramatically, with contemporary studies revealing rates ranging from 2 to 4% [13]. IVL uses sonic pressure waves that fragment circular vascular calcifications, which subsequently require less aggressive dilatation, thus reducing the risk for vascular complications such as dissections and perforations and explaining the lower risk of complications that we saw in our study (IVL: 7.2% vs. PTA: 13.7%) [7,14]. However, these rates are still significantly higher than those in patients undergoing TAVI without PAD.

Landmark randomized controlled trials studying intermediate-risk TAVI patients without PAD have reported similar rates of all-cause mortality at 3 years (~19%) irrespective of the use of self-expanding or balloon-expandable valves [15]. In our study, 3-year all-cause mortality rates ranged from 34.9% to 38.6% irrespective of choice of treatment between PTA or IVL. In contrast, Ullah et al. found no difference in 30- and 180-day mortality rates between TAVI patients with and without PAD [16]. This discrepancy may be due to the severity of PAD in our population. The increased mortality risk in PAD patients undergoing TAVI necessitating additional intervention (i.e., PTA, IVL) is not accurately reflected by current standard risk stratification tools (i.e., STS, Euroscore). The Hostile Score is the only known tool that allows stratification of outcomes in PAD patients undergoing TAVI.

Data on stroke/TIA rates based on the type of intervention (PTA or IVL) is currently limited. Our study showed that PAD patients undergoing TAVI via TFA had a 2.0–2.4% rate of stroke/TIA at 30-day follow-up irrespective of treatment with PTA or IVL. This rate is similar to those reported in contemporary studies in the general population without PAD [4,17].

The length of hospital stay after the index procedure was significantly longer in the PTA group compared to the IVL group (7.06 ± 6.69 vs. 4.29 ± 4.78 days; *p* < 0.001; 95% CI: 1.63–3.91). Both groups had longer stays than standard TAVI patients without PAD [18]. Due to their increased complexity and higher risk of complications, these patients should probably be referred to higher-volume and more experienced centers.

At 1-year follow-up, rates of the composite of all-cause mortality, stroke/TIA, and major vascular complications were significantly higher in patients treated with PTA compared to those treated with IVL (32.1% vs. 23.9%, *p* = 0.023). This difference was driven by higher rates of major vascular complications in the PTA group (Figure 5). According to our Cox regression analysis, the higher the Hostile Score, the worse the outcome, with an increased rate of the composite of all-cause mortality major bleeding, major vascular complications, and stroke/TIA irrespective of treatment with PTA or IVL. The Hostile Score is a valuable tool for risk stratification of PAD patients undergoing TAVI and may aid in decision-making and procedural planning. However, given the observational design, the influence of operator discretion and center-specific protocols, and significant baseline differences—particularly the lower rate of ultrasound-guided femoral access in the PTA group—our findings should be interpreted as hypothesis-generating rather than conclusive. The Hostile Score should be further validated to ensure the consistency of these results.

While the dichotomized Hostile Score demonstrated clinical utility in stratifying risk, modeling the score as a continuous variable did not yield significant associations with key outcomes, suggesting its predictive value may be threshold-dependent or limited by non-linear effects.

## 5. Study Limitations

This study is a sub-analysis of a larger observational study. This retrospective, non-randomized design is inherently limited by potential selection bias and residual confounding, despite our efforts to adjust for known covariates. Despite adjusting for key covariates such as all-cause mortality, stroke/TIA, and major adverse events (MAE), unmeasured confounders may still exist. Data on frailty indices and other variables potentially influencing outcomes were not available. The larger Hostile registry was not randomized; therefore, its findings should be viewed as hypothesis-generating. Data on the use of PTA alongside IVL were not collected, leaving unknown the proportion of IVL patients who also underwent PTA versus those who did not; this precludes the assessment of combined or staged therapy effects. Furthermore, the study focused on patients with severe obstructive PAD and excluded those with aortic aneurysm, thrombus, or those who underwent PTA as a bailout procedure for iatrogenic complications. Including these patients would have confounded comparisons across access routes. VARC-3 major vascular complications encompass a diverse category of events not individually captured by this study. The Hostile Score, based on anatomical variables collected at each participating center, was not adjudicated by a central core laboratory. Treatment modality (PTA vs. IVL) was selected at the discretion of the operator and may reflect center-level expertise or preference. Ultrasound-guided femoral access was notably underutilized in the PTA group (38.3%), which could have influenced complication rates. Finally, ethnicity data were not collected as part of the registry, limiting the ability to assess differences in outcomes across racial or ethnic subgroups. The study population was predominantly European, which may limit the generalizability of findings to more diverse populations.

## 6. Conclusions

In patients with severe PAD undergoing TAVI, PTA treatment was associated with higher 30-day rates of major bleeding and major vascular complications than IVL treatment without differences in mortality rates over a 3-year follow up period. The Hostile Score may be an additional tool for risk stratification of PAD patients undergoing TAVI, and its utility should be validated in a larger trial.

## Figures and Tables

**Table 1:** Population characteristics.

Characteristic	PTA	IVL	*p*-Value
	(*n* = 352)	(*n* = 166)	
Age	81.1 ± 6.4	79.6 ± 7.0	0.051
Male	184 (52.3)	93 (56)	0.451
Hypertension	316 (90.0)	149 (89.8)	1
Diabetes mellitus	116 (33.0)	59 (35.5)	0.619
CAD	246 (69.9)	110 (66.3)	0.418
Prior stroke/TIA	41 (11.6)	8 (4.8)	0.015
Prior PCI	115 (32.7)	74 (44.6)	0.925
Prior MI	99 (28.1)	42 (25.3)	0.527
Prior CABG	78 (22.2)	39 (23.5)	0.737
Atrial fibrillation	135 (38.4)	60 (36.1)	0.698
Baseline serum creatinine	1.48 ± 1.37	1.41 ± 1.1	0.586
LVEF	52.9 ± 11.8	52.6 ± 11.8	0.768
BMI	25.7 ± 4.6	25.6 ± 5.7	0.882
NYHA class 1 to 4	2.7 ± 0.7	2.7 ± 0.6	0.7
EuroSCORE II	7.9 ± 6.8	8.1 ± 6.5	0.797
Hostile Score	7.1 ± 2.5	7.1 ± 2.6	0.877
STS-PROM	6.1 ± 4.4	5.6 ± 3.2	0.151

Values are mean ± SD or n/N (%). BMI = body mass index; CABG = coronary artery bypass graft; CAD = coronary artery disease; LVEF = left ventricular ejection fraction; MI = myocardial infarction; NYHA = New York Heart Association; PCI = percutaneous coronary intervention; STS-PROM = Society of Thoracic Surgeons Predicted Risk of Operative Mortality.

**Table 2:** Procedural characteristics.

Characteristic	PTA	IVL	*p*-Value
	(*n* = 352)	(*n* = 166)	
Surgical cutdown	48 (13.6)	9 (5.4)	0.003
Percutaneous access	304 (86.4)	157 (94.6)	0.451
Ultrasound-guided femoral puncture	128 (38.3)	107 (81.1)	<0.001
**Type of valve**			
Evolut/CoreValve	133 (37.8)	64 (38.6)	
Sapien	158 (44.9)	64 (38.6)	
Other	55 (15.6)	37 (22.3)	
Not implanted	2 (0.6)	0 (0)	
**Closure device**			
Angioseal	4 (1.1)	0 (0)	
MANTA	14 (4.0)	15 (9.0)	
ProGlide	237 (67.3)	127 (76.5)	
Prostar	20 (5.7)	7 (4.2)	
Surgical	44 (12.5)	0 (0)	
Combination of devices	20 (5.7)	8 (4.8)	

Values are *n*/N (%).

## Data Availability

The original contributions presented in this study are included in the article. Further inquiries can be directed to the corresponding author(s).

## Appendix A

**Table A1:** Multivariable logistic regression analysis including access type and closure device as covariates.

Variable	Log(OR)	Std. Error	z	*p*-Value	OR	95% CI Lower	95% CI Upper
IVL	−0.488	0.288	−1.697	0.09	0.614	0.349	1.079
PTA	11.78	565.135	0.021	0.983	130,674.63	0.0	∞
Closure Device: Combination of devices	0.446	1.302	0.342	0.732	1.561	0.122	20.022
Closure Device: MANTA	−2.03	1.611	−1.26	0.208	0.131	0.006	3.089
Closure Device: ProGlide	−0.626	1.245	−0.503	0.615	0.535	0.047	6.132
Closure Device: Prostar	−0.608	1.329	−0.458	0.647	0.544	0.04	7.358
Closure Device: Surgical	10.765	565.137	0.019	0.985	47,357.814	0.0	∞
